# A Novel Hybrid Swarm Optimized Multilayer Neural Network for Spatial Prediction of Flash Floods in Tropical Areas Using Sentinel-1 SAR Imagery and Geospatial Data

**DOI:** 10.3390/s18113704

**Published:** 2018-10-31

**Authors:** Phuong-Thao Thi Ngo, Nhat-Duc Hoang, Biswajeet Pradhan, Quang Khanh Nguyen, Xuan Truong Tran, Quang Minh Nguyen, Viet Nghia Nguyen, Pijush Samui, Dieu Tien Bui

**Affiliations:** 1 Faculty of Information Technology, Hanoi University of Mining and Geology, No. 18 Pho Vien, Duc Thang, Bac Tu Liem, Hanoi 10000, Vietnam; Ngothiphuongthao@humg.edu.vn (P.-T.T.N.); Nguyenquangkhanh@humg.edu.vn (Q.K.N.); 2 Faculty of Civil Engineering, Institute of Research and Development, Duy Tan University, Da Nang 550000 Vietnam; hoangnhatduc@dtu.edu.vn; 3 Centre for Advanced Modelling and Geospatial Information Systems (CAMGIS), Faculty of Engineering and IT, University of Technology Sydney, Sydney, NSW 2007, Australia; biswajeet24@gmail.com; 4 Department of Energy and Mineral Resources Engineering, Choongmu-gwan, Sejong University, 209 Neungdong-ro, Gwangjin-gu, Seoul 05006, Korea; 5 Faculty of Geomatics and Land Administration, Hanoi University of Mining and Geology, No. 18 Pho Vien, Duc Thang, Bac Tu Liem, Hanoi 10000, Vietnam; Tranxuantruong@humg.edu.vn (X.T.T.); Nguyenquangminh@humg.edu.vn (Q.M.N.); Nguyenvietnghia@humg.edu.vn (V.N.N.); 6 Geographic Information Science Research Group, Ton Duc Thang University, Ho Chi Minh City 700000, Vietnam; 7 Faculty of Environment and Labour Safety, Ton Duc Thang University, Ho Chi Minh City 700000, Vietnam; 8 Geographic Information System Group, Department of Business and IT, University of South-Eastern Norway, N-3800 Bø i Telemark, Norway; Dieu.T.Bui@usn.no

**Keywords:** flash floods, Sentinel-1, GIS, artificial neural network, firefly algorithm, Levenberg–Marquardt backpropagation

## Abstract

Flash floods are widely recognized as one of the most devastating natural hazards in the world, therefore prediction of flash flood-prone areas is crucial for public safety and emergency management. This research proposes a new methodology for spatial prediction of flash floods based on Sentinel-1 SAR imagery and a new hybrid machine learning technique. The SAR imagery is used to detect flash flood inundation areas, whereas the new machine learning technique, which is a hybrid of the firefly algorithm (FA), Levenberg–Marquardt (LM) backpropagation, and an artificial neural network (named as FA-LM-ANN), was used to construct the prediction model. The Bac Ha Bao Yen (BHBY) area in the northwestern region of Vietnam was used as a case study. Accordingly, a Geographical Information System (GIS) database was constructed using 12 input variables (elevation, slope, aspect, curvature, topographic wetness index, stream power index, toposhade, stream density, rainfall, normalized difference vegetation index, soil type, and lithology) and subsequently the output of flood inundation areas was mapped. Using the database and FA-LM-ANN, the flash flood model was trained and verified. The model performance was validated via various performance metrics including the classification accuracy rate, the area under the curve, precision, and recall. Then, the flash flood model that produced the highest performance was compared with benchmarks, indicating that the combination of FA and LM backpropagation is proven to be very effective and the proposed FA-LM-ANN is a new and useful tool for predicting flash flood susceptibility.

## 1. Introduction

Floods are considered as one of the major natural disasters in the world, in terms of human casualties and financial losses [1,2]. Among several types of floods, flash floods are typically disastrous and are distinguished from regular floods by their rapid occurrence on short timescales, i.e., less than six hours [3]. Flash flood hazards are often triggered by heavy downpours, torrential rainfalls, or tropical rainstorms. Reports on the destructive effects of flash floods on human lives have been observed worldwide [4,5,6,7,8,9]. Human factors also contribute to the occurrence of flash floods i.e., deforestation and unplanned land use. Deforestation obviously weakens the capability of flood prevention because forests significantly reduce water surface runoff and transfer the excess water into the groundwater and aquifers [10], In addition, the population growth leads to the fact that many newly built settlements are located in areas susceptible to floods.

Due to the devastating economic, environment, and social aspect effects of flash floods, many studies have been dedicated to spatial modeling of floods and establishing flood susceptibility maps at a regional scale [11,12,13,14]. This is because the determination of flood-prone areas is an essential step in the prevention and management of future floods [15,16]. Nevertheless, the construction of flash flood susceptibility maps is a difficult task, especially in large areas, because flash floods are complicated processes which have region-dependent features and occur nonlinearly across a variety of spatio-temporal scales [17].

In recent years, the rapid advancement of Geographic Information System (GIS), remote sensing, and machine learning have given scientists effective tools for dealing with the complexity of spatial flood modeling [18,19,20]. The spatial data extracted from GIS greatly enhances the understanding and the assessment of flood risks for the whole region under analysis. Moreover, these GIS-based datasets can be combined with modern machine learning approaches to construct powerful tools for spatial prediction of floods. New remote sensing sensors i.e., Sentinel-1A and B, provide new tools for flood detection and mapping with high accuracy [21,22]. Machine learning methods with their capabilities dealing with nonlinear and multivariate data have proven their usefulness in establishing flood susceptibility maps in various countries around the world [23].

Moreover, recent reports with positive results of machine learning applications in solving the problem of interest have been observed extensively in the literature. This is because machine learning has the ability to explore complicated relationships between factors in various real-world problems [24,25]. For flood modeling, Nandi, et al. [26] constructed a flood hazard map in Jamaica based on logistic regression and principal component analysis. A GIS-based flood susceptibility assessment and mapping using frequency ratio and weights-of evidence bivariate statistical models have been put forward by Khosravi, et al. [27]. Tien Bui, Pradhan, Nampak, Bui, Tran and Nguyen [15] and Razavi Termeh, et al. [28] proposed novel data-driven methods based on artificial intelligence optimized by metaheuristic algorithms for flood susceptibility. Lee, et al. [29] investigated the applicability of boosted-tree and random forest techniques for flood susceptibility prediction in a metropolitan city. A probabilistic model based on Bayesian framework for spatial prediction of floods has been proposed by Tien Bui and Hoang [30]. Chapi, et al. [31] combined a bagging algorithm and a logistic model tree to create a new tool for flood susceptibility mapping. Sachdeva, et al. [32] recently incorporated GIS, support vector machine and a swarm optimization algorithm to formulate a flood risk assessment model applied in India. Rahmati and Pourghasemi [33] analyzed the spatial data and identified critical flood prone areas with the help of various techniques including the evidential belief function and the classification trees.

Among machine learning methods, artificial neural networks (ANNs) are perhaps some of the most extensively used in flood modeling [34,35] as well as spatial predictions of other natural hazards [36,37,38,39]. This method possesses a strong capability in analyzing nonlinear and multivariate data as well as the ability of universal modeling. Despite these advantages, the application of ANNs in GIS-based modeling of flash flood susceptibility is still limited. In addition, previous works applying ANN in spatial modeling of natural hazards often resorted to gradient-based algorithms with backpropagation as a conventional way for training the models. This conventional approach updates the weights of an ANN model to minimize the prediction errors during the training phase. Although gradient-based algorithms with backpropagation are fast, this training method suffers from the risk of being trapped in local minima, especially in a multi-modal error space [40]. This disadvantage significantly deteriorates the predictive capability of ANN-based flash flood prediction models.

To counteract the aforementioned limitation of gradient-based algorithms, metaheuristics as a global searching method have been employed to improve the ANN training phase. Various metaheuristic algorithms, such as cuckoo search optimization [41], bat optimization [42], monarch butterfly optimization [43], shuffled frog leap algorithm [44], kidney-inspired algorithm [45], and an improved particle swarm optimization [46], have been recently proposed and investigated. Previous studies show improved performances of metaheuristic-assisted models compared to the traditional models. A review by Ojha, et al. [47] pointed out an increasing trend of applying metaheuristics as a tool for ANN models’ construction phase.

The construction of an ANN model involves the optimization of connecting weights; in addition, the landscape of the error function can be highly complicated with numerous local minima. These facts entail that the stochastic search of metaheuristic must involve the cooperation of a considerable number of searching agents (also called population members). The search space exploration of such searching agents typically represents a huge computational burden and has a slow convergence rate. Metaheuristic algorithms often require a large amount of function during the optimization of the ANN models ‘weights. Therefore, it is necessary to combine the advantages of both metaheuristic and gradient-based algorithms to come up with an effective method for ANN model training.

This study puts forward a novel method, which employs gradient-based algorithm of Levenberg-Marquardt backpropagation and the metaheuristic firefly algorithm algorithm. In this integrated framework, the firefly algorithm acts as a global search engine and the backpropagation algorithm plays the role of a local search with the aim of accelerating the optimization process. To train and verify the new ANN model used for flash flood susceptibility mapping, the Bac Ha Bao Yen (BHBY) area in the northwestern region of Vietnam was selected as a case study. This area belongs to a region which is highly susceptible to flash flooding occurrences due to its relief characteristics, i.e., rough and steep terrains [10]. Reports on the losses of human lives after the occurrences of flash floods in this area are regular news in the mass media. For instance, in August 2017, flash floods isolated many towns in this region and killed 18 people [48].

## 2. Background of the Methods Used

### 2.1. Flash-Flood Detection from Multitemporal Sentinel-1A SAR Imagery

Spatial prediction of areas prone to flash flooding using machine learning requires understanding and learning from events occurred in the past and present [30,49]; therefore, establishment of flash-flood inventory map is a key issue and mandatory task. A literature review points out that mapping of flash flood inventories is still the most critical task in the literature because flash floods are usually characterized both by short temporal and spatial scales that are difficult to observe and detect [49]. Optical images are not suitable because they are sensitive to illumination and bad weather conditions [22]. Most of published works collected flash-flood event data using handheld GPS devices and field surveys, which consume both time and cost, i.e., in [16,20].

In this research, Sentinel-1A SAR imagery is used for deriving flood inventories. Sentinel-1A is a satellite launched on 3 April 2014 by the Europe Space Agency (ESA) in the Copernicus Programme [50]. The mission has a repeat cycle of 12 days providing C-band SAR data (wavelength 3.75–7.5 cm, frequency 4–8 GHz) in four acquisition modes, interferometric wide-swath (IW), extra wide-swath (EW), wave mode (WV), and strip map (SM). Although Sentine-1A provides two dual-polarized data sources, co-polarized vertical transmit/vertical receive (VV) and cross-polarized vertical transmit/vertical receive (VH); however, the VV data provides better results [51,52], therefore it was selected for flash flood detection in this study. Accordingly, four images (Table 1) were acquired in IW mode (250 km swath width and 10-m resolution), Level-1 ground range detected (GRD) format, and ascending direction.

**Figure 1:**
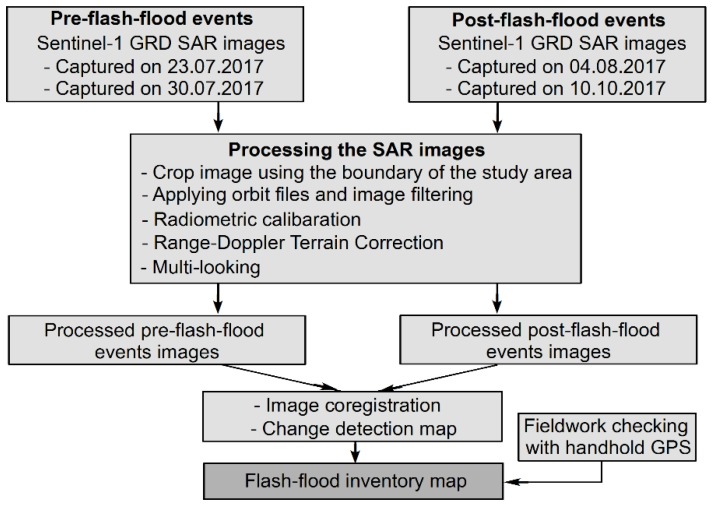
Methodological flow chart for flash-flood detection using the multi-temporal Sentinel-1 SAR images.

The proposed methodological approach to obtain flash-flood inventories for the study area using Sentinel-1A SAR imagery is shown in Figure 1. This approach uses the concept of change detection that requires image pairs captured pre- and post-flash flood events and the same satellite track. The processing of the Sentinel-1 GRD imagery consists of the following main tasks: (1) updated satellite position and velocity information using the precise orbit files, and then, the Lee filter [53] and multi-looking were applied to remove the speckle in these images; (2) Radiometric calibration was used to remove radiometric bias and ensure values at pixels are the real backscatter of the reflecting surface; (3) Range-Doppler terrain correction was applied using shuttle radar topography mission digital elevation model (SRTM DEM) to remove images distortions and re-projected the resulting images to the UTM 48N projection of the study area.

**Figure 2:**
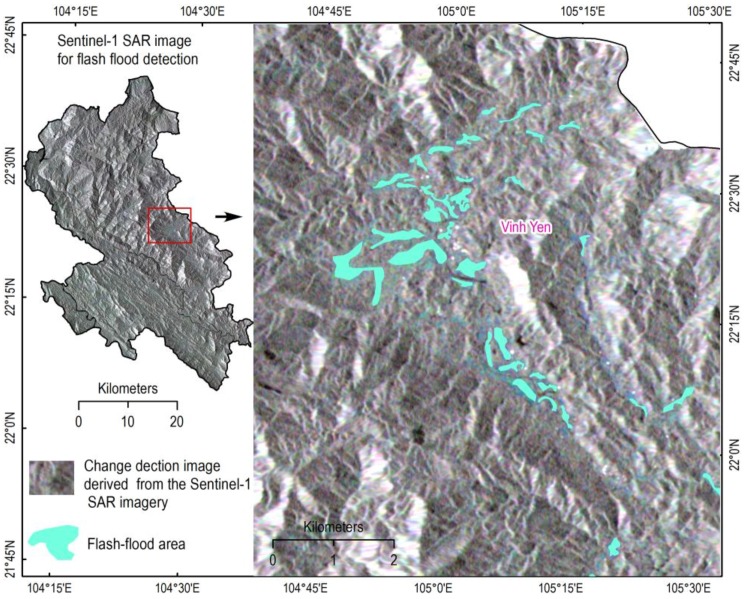
Flash flood areas detected from the Sentinel-1 SAR images.

Once the processing phase of these images were completed, co-registration between the pre-flash flood and post-flash flood images were performed, and subsequently, flash flood areas were detected. These flood areas were manually digitalized using ArcGIS. Finally, these flash flood results were randomly checked in the fieldwork phase using handhold GPS. Figure 2 shows flash flood areas detected by the above Sentinel-1A SAR imagery.

### 2.2. Artificial Neural Network for Flash Flood Modeling

**Figure 3:**
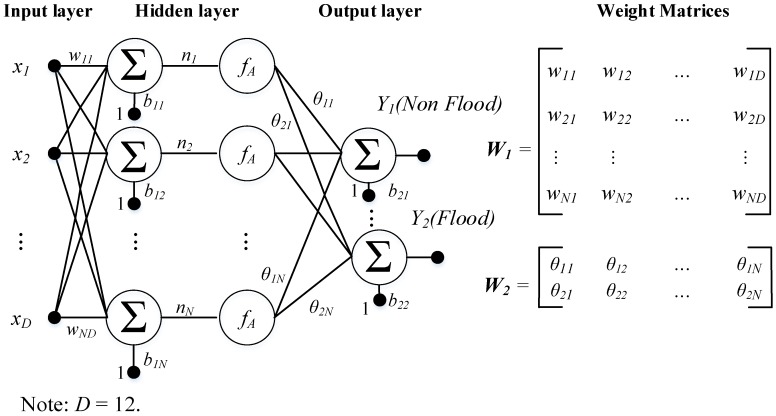
The structure of an ANN model used for spatial prediction of flash flood.

A multilayer artificial neural network (ANN) is a supervised machine learning algorithm which imitates the characteristics of actual biological neural networks [54]. An ANN can be trained with input data (flash flood conditioning factors) with ground truth labels (flash-flood and non-flash-flood); the trained ANN model is then used to predict the output class labels of flash flood occurrences. Generally, the structure of an ANN is arranged into three connected layers: input, hidden, and output (see Figure 3). The first layer contains neurons, which are flash flood conditioning factors. The second layer, including individual neurons, perform the task of information processing to yield the class labels of flood susceptibility in the output layer.

The aim of training flash flood prediction model is to determine a mapping function $f:X\in R^{D}\to Y^{C}$ $f:X\in R^{D}\to T\in R^{C}$ where *D* denotes the number of input flash flood factors and *C* = 2 is the two output classes, no flood (*C*_1_ = −1) and flood (*C*_2_ = +1). The mapping function *f* can be briefly described in the following form [55]:

(1) $$\begin{array}{l} Y_{1}=f_{1}(X)=b_{21}+W_{2}\times (f_{A}(b_{1}+W_{1}\times X))\\ Y_{2}=f_{2}(X)=b_{22}+W_{2}\times (f_{A}(b_{1}+W_{1}\times X)) \end{array}$$

where *W*_1_ and *W*_2_ are two weight matrices (see Figure 3). *b*_1_ = [*b*_11_ *b*_12_ … *b*_1N_] and *b*_2_ = [*b*_21_ *b*_22_] are bias vectors; *f_A_* denotes the log-sigmoid activation function given as follows:

(2) $$f_{A}(n_{j})=\frac{1}{1+\exp(-n_{j})},$$

where *j* = 1, 2, …, *N*.

In the ANN learning phase, the weight matrices and the bias vectors are adapted via the framework of error backpropagation [56]. The Mean Square Error (MSE) is used as objective function as follows:

(3) $$MSE=\underset{W_{1},W_{2},b_{1},b_{2}}{\min}\frac{1}{M}\sum_{i=1}^{M}er_{i}^{2},$$

where *M* is the total number of the samples in the training set; *er_i_* is output error; *er_i_* = *Y_i,P_* − *Y_i,A_*; *Y_i,P_* and *Y_i,A_* are predicted and actual values, respectively.

Notably, for not large data sets, the Levenberg–Marquardt algorithm (LM) [57,58] is a suitable method for training ANN structures. The advantage of the LM method is recognizable through its fast and stable convergence [59]. In this approach, the weights of an ANN model can be adapted by Equation (4) [57]:

(4) $$\;w_{\left(i+1\right)}=w_{i}-{\left(J_{i}^{T}J_{i}+\lambda I\right)}^{-1}J_{i}^{T}er_{i},\;$$

where *J* denotes the Jacobian matrix; *I* represents the identity matrix; *λ* is the learning rate parameter.

### 2.3. Firefly Algorithm (FA) for Optimizatizing Flash Flood Model

FA is a swarm-based algorithm proposed by Yang [60], which was inspired by the flashing communication of fireflies. The pattern of firefly flashes is unique where each firefly in the swarm is attracted to brighter ones, and meanwhile, it explores and searches for prey randomly. FA is considered as a global optimization method, in which, an advanced swarm intelligence is used to search and find the best solution, effectively [61]. Thus, FA has proven as a highly suitable tool for dealing with complex optimization problems in continuous space, including the problem of neural network training [62,63]. Recent studies have shown excellent performances of FA when applied in various domains [64,65,66,67]. In general, the FA method utilizes the following rules [68]:

- All fireflies of a swarm are unisex; therefore, a firefly will be attracted to other fireflies without paying attention to their sex.
- The attractiveness degree of a firefly is directly related to its brightness. The attractiveness will be decreased when the distance is increased. If no bright signal is received from other fireflies, the firefly will move randomly.
- The brightness of a firefly is determined intern of cost function.

**Figure 4:**
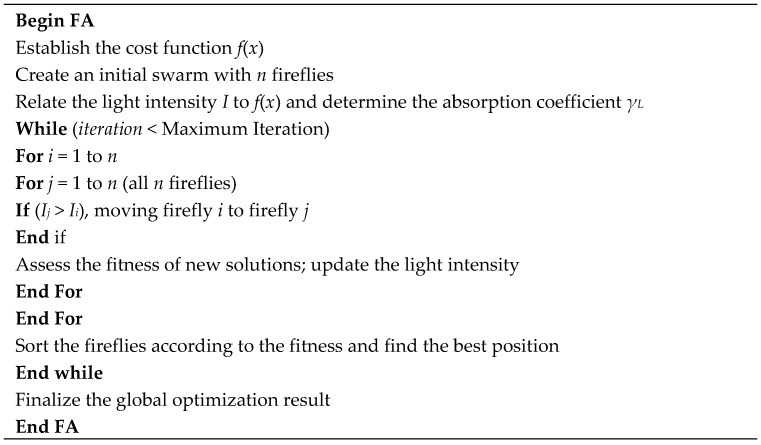
The FA used for global optimization.

The FA pseudo code is illustrated in Figure 4 below:

The light intensity *I(r)* is computed using Equation (5) as follows:

(5) $$I(r)=I_{o}\exp(-\gamma_{L}r^{2}),$$

where *I_o_* represents the light intensity of the firefly source; *γ_L_* is the light absorption coefficient; and *r* denotes the distance from the firefly source.

The attractiveness degree *β* of a firefly in the swarm is estimated using Equation (6):

(6) $$\beta =\beta_{o}\exp(-\gamma_{L}r^{2}),$$

Distance of any two fireflies *x_i_* and *x_j_* in the swarm in dimensional space (D) is defined using Equation (7) as follows:

(7) $$r_{ij}=\left\|x_{i}-x_{j}\right\|=\sqrt{\sum_{k=1}^{D}{\left(x_{i,k}-x_{j,k}\right)}^{2}},$$

When a specific firefly *x_i_* gets bright signal from firefly *x_j_*, it will move to the *i*th firefly using Equation (8) below:

(8) $$x_{i}=x_{i}+\beta_{o}\exp(-\gamma_{L}r_{ij}^{2})(x_{i}-x_{j})+\alpha (\omega -0.5),$$

where *γ_L_* is the light absorption coefficient; *β*_0_ is the attractiveness at *r_ij_* = 0; α denotes a trade-off constant; and ω is a random number deriving from the Gaussian distribution.

## 3. The Study Site and the GIS Database

### 3.1. Study Area

**Figure 5:**
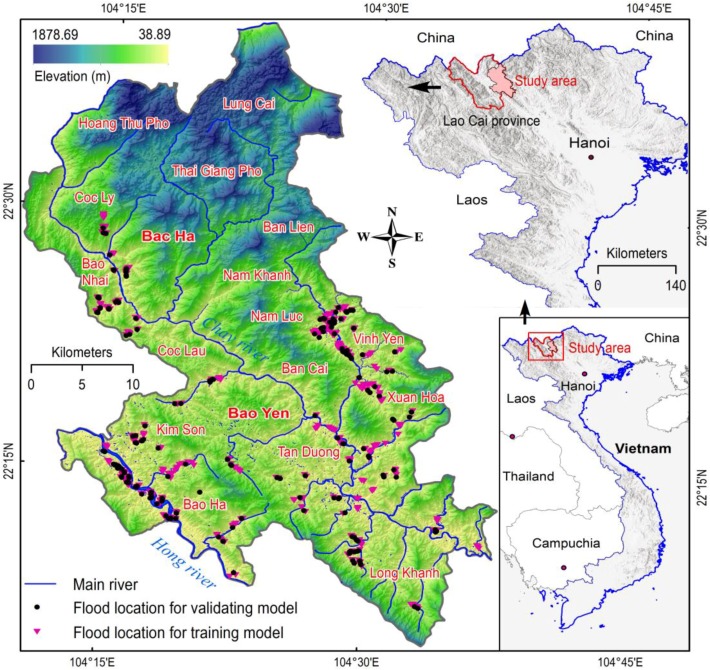
Location of the study area and flood locations.

The study area (see Figure 5) covers two districts—Bac Ha and Bao Yen (BHBY)—which belong to Lao Cai Province in the northwestern area of Vietnam. BHBY occupies an area of about 1510.4 km^2^, between longitudes of 104°10′ E–105°37′ E and latitudes of 22°5′ N–22°40′ N. The altitude ranges between 38.9 m at the river valleys to 1878.69 m above sea level at the mountain range of Bac Ha. This is typically a mountainous region with a complex network of rivers. Two main rivers flow in the study area, the Hong River and Chay River. The first one, which bisects the province and flows through the study area with a length of approximately 28.7 km is the biggest river. The second one is the major river flowing from north to south, with an estimated length of 91.6 km.

Since the BHBY is a typical mountainous area, it has a cold-dry climate, which often lasts from October to March. The other months from April to September correspond to the rainy season. According to the Lao Cai statistical yearbook from 2010–2016 (measured at the Bac Ha station) [69], monthly rainfall varied from 9.0 mm (March 2010) to 540 mm (August 2016) and the total rainfall per year was from 1280.2 mm (2015) to 1844.9 mm (2016). More than 80% of the total rainfall per year was received in the rainy season. The rainfall is concentrated especially in three months (June to August), with the total rainfall of these three months accounting for more than 50% of the yearly rainfall [69]. For the period of 2010–2016, the annual average temperature varied from 19.27 °C and 23.77 °C with the lowest monthly temperature being 10.6 °C in January 2014 (measured at the Bac Ha station) and the highest monthly temperature was 29.5 °C in June 2015 (measured at the Bao Yen station) [69].

Total population of the study area is 145,208 people in 2017 [69] and they mainly belong to ethnic minority groups that are highly vulnerable to natural hazards, especially flash floods, due to population growth and deforestation [70]. For instance, recent severe and torrential rainstorms caused by a tropical depression occurred on October 2017 in northern Vietnam (including the study area) created widespread flash floods and destroyed more than 16,000 houses.

### 3.2. Flood Inventory Map and Conditioning Factors

Prediction of flash-flood prone areas in this research is based on a statistical assumption that future-flash flooding areas are governed by the same conditions which generated flash-flooded zones in the present and the past [30]. Therefore, flash-flood inventories and their geo-environmental conditions (i.e., topological, climatic, and hydrological characteristics) in the past and present must be extensively studied and collected [20,28].

In this research, the flash-flood inventory map with 654 flash flood polygons was used (see Figure 5). The map was constructed based on the change detection of the Sentinel-1A SAR imagery as mentioned in Section 2.1. Although the data for this study is from 2017, however, flash floods are recurrent events; therefore, severe flash flood locations in the BHBY area were revealed.

The next step is to determine flash-flood influencing factors, a crucial task. Literature review shows that it is still no consensus on which factors must be used, and in general, factors should be selected based on flash-flood characteristics and the availability of geospatial data in the study areas [28,71]. Accordingly, a total of 12 conditioning factors were considered in this study: elevation (IF1), slope (IF2), aspect (IF3), curvature (IF4), topographic wetness index (TWI) (IF5), stream power index (SPI) (IF6), toposhade (IF7), stream density (IF8), rainfall (IF9), normalized difference vegetation index (IF10), soil type (IF11), and lithology (IF12).

**Figure 6:**
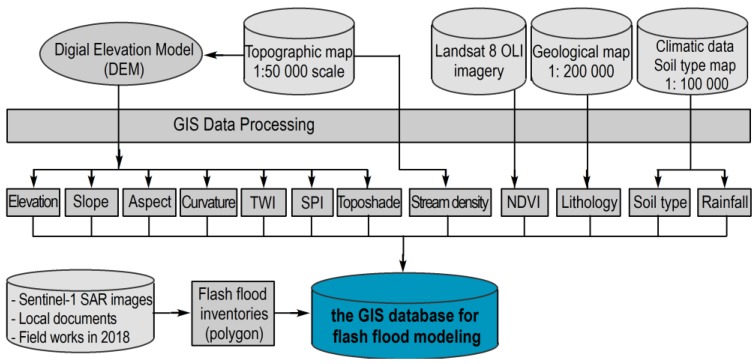
The established GIS database for the flash-flood modeling.

**Figure 7:**
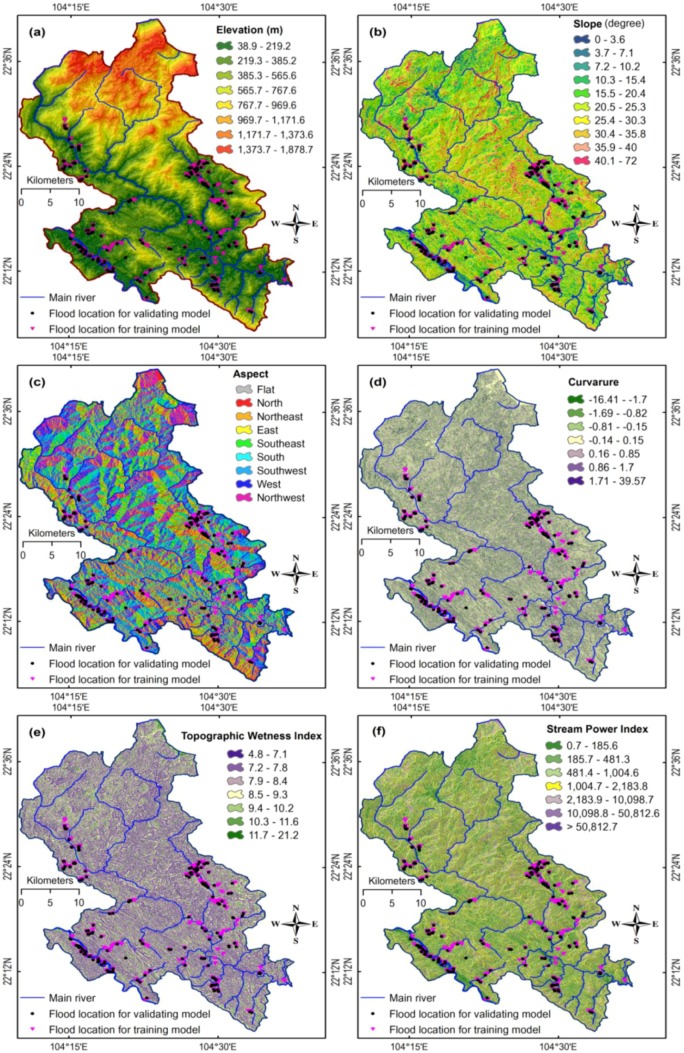

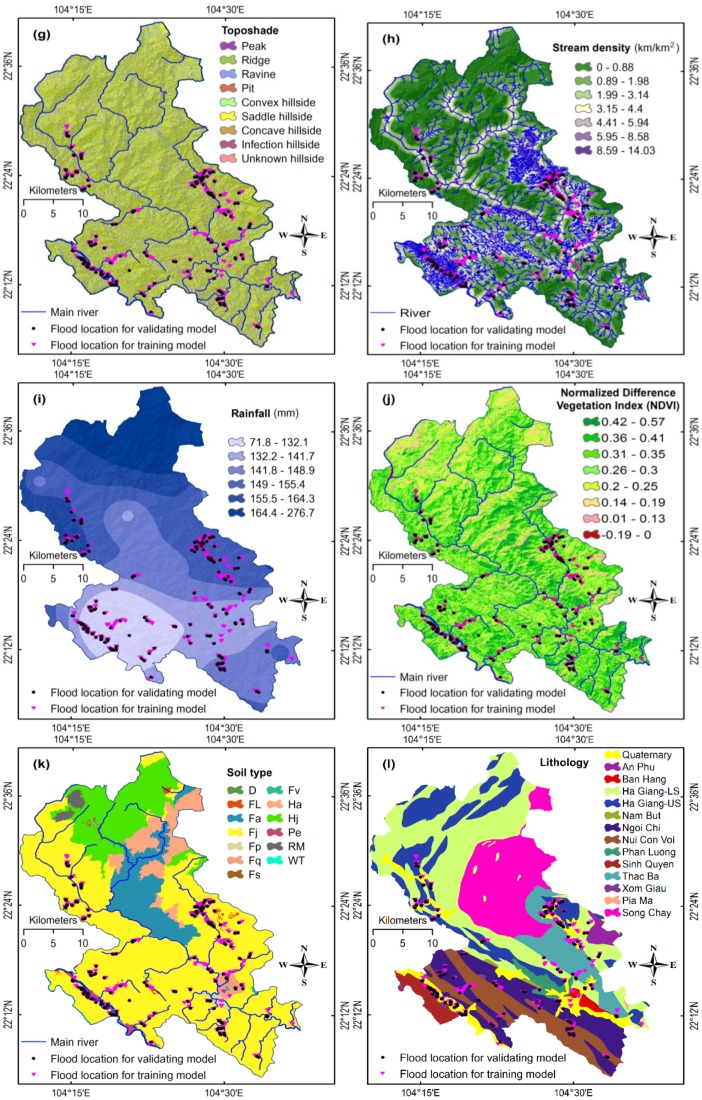
Flash flood conditioning factors: (**a**) elevation; (**b**) slope; (**c**) aspect; (**d**) curvature; (**e**) Topographic Wetness Index; (**f**) Stream Power Index. Flash flood conditioning factors: (**g**) toposhade, (**h**) stream density; (**i**) rainfall; (**j**) Normalized Difference Vegetation Index; (**k**) soil type; and (**l**) lithology.

To prepare data for flash-flood modeling, a GIS database (see Figure 6) was established, which contains historical flash-flood events in 2017, topographic maps and their features, Landsat 8 imagery (30 m resolution, acquired on 20 December 2017 [72]), geology, and total rainfall in October 2017 at measure stations in and around the study area are acquired. The schematic maps of the 12 factors are shown in Figure 7. These factors were processed using ArcGIS 10.4 and IDRISI Selva 17.01.

Next, a Python tool was programed by the authors to generate the flash-flood susceptibility map in the form of the indices produced by the flash-flood model in the ArcGIS environment. The compiled inventory database includes two class outputs: “flood” and “non-flood”. As stated above, in this study, 654 flood locations have been recorded; therefore, 654 data samples of the “flood” label are extracted from the flood inventory map. Because flash-flood modeling in this research is based on machine learning classification, which is different to that of traditional flood modeling approaches; therefore, 654 data samples of non-flood areas are randomly generated from not-yet flood areas [73]. Herein, equal proportion of the samples is suggested to use for avoiding bias [73,74,75]. Consequently, a total of 1308 data samples are derived.

## 4. The Proposed Metaheuristic-Optimized Neural Network Model for Flash Flood Susceptibility Prediction

This section provides description of the proposed flash flood prediction model that integrates the ANN machine-learning model and the FA metaheuristic approach improved by the Levenberg–Marquardt (LM) algorithm. The hybrid method of FA and LM, denoted as FA-LM, is proposed as the method for training the ANN model. After being trained, the FA-LM trained ANN, denoted as FA-LM-ANN, can assign class labels (either non-flash flood or flash flood) to each input information containing the aforementioned 12 conditioning factors.

**Figure 8:**
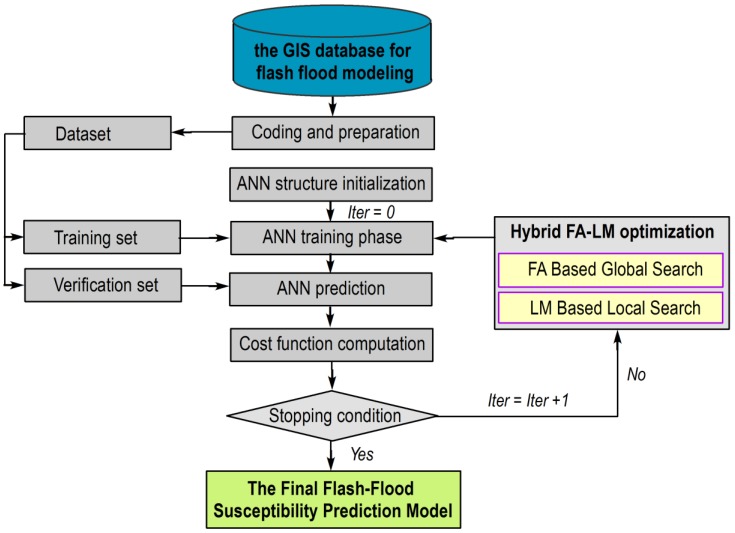
The proposed metaheuristic-optimized neural network model for flash flood susceptibility prediction.

The overall structure of the proposed model is depicted in Figure 8.

### 4.1. Encoding the ANN Structure for Flash Flood Modeling

The structure of an ANN model is generally determined by its weight matrices *W*_1_ and *W*_2_. The size of the matrix *W*_1_ is *N_R_* × *N_I_* + 1 where *N_R_* and *N_I_* denote hidden neurons and input neurons, respectively. It is noted that the number of column of *W*_1_ is *N_I_* + 1 to include a vector of bias. In this analysis, *N_I_* = 12 which is the number of flash flood conditioning factors. The number of neurons in the hidden layer should be large enough to facilitate the learning and inferring complex mapping functions. However, the value of *N_R_* should not be too large since the resulting ANN model can be difficult to train and exceedingly complex model is highly susceptible to overfitting.

According to the recommendation of Heaton [76], *N_R_* is roughly set to be *N_R_* = 2*N_I_*/3 + *N_O_*, where *N_I_* = 12 (flash flood conditioning factors) and *N_O_* = 2 (output or flood susceptibility). Moreover, a value of *N_R_* that exceeds 1.5 × *N_I_* often results in longer training time without significant improvements in predictive accuracy. Based on such suggestions and several trial-and-error runs, *N_R_* for the ANN trained with the collected data set is chosen to be 9. Moreover, the size of the matrix *W*_2_ is *N_O_* × *N_R_* + 1. Notably, it is required that a solution of the FA-LM algorithm is coded in forms of a vector. Hence, the two matrices *W*_1_ and *W*_2_ are vectorized and then concatenated to construct a solution. Accordingly, the total number of decision variables optimized by the FA-LM optimization is estimated as *N_R_* × (*N_I_*+ 1) + *N_O_* × (*N_R_*+ 1) and equal to 137.

### 4.2. Proposed Cost Function for Flash-Flood Modeling

During the searching process of the FA-LM optimization, to exhibit the appropriateness of each solution, a cost function must be defined. The cost function (CF) of the FA-LM algorithm is given as follows:

(9) $$CF_{}=\frac{MSE_{TR}+MSE_{VA}}{2},\;$$

where *MSE_TR_* and *MSE_VA_* denote the mean squared error (MSE) for the training dataset (80% of the total model construction samples) and the validating dataset (20% of the total model construction samples), respectively. The rationale of the cost function described in Equation (9) is to guide the FA-LM searching process to minimize the prediction error for both the training dataset and the validating dataset. The reason for the inclusion of validating data sample in the calculation of the cost function is to alleviate overfitting. It is noted that overfitting happens when the constructed model has a very good performance on the training set; however, its performance when predicting novel input data is very poor. Thus, it is important that the ANN model have good prediction accuracy in both training set and validating set.

### 4.3. The FA-LM Algorithm: A Hybridization of Metaheuristic Optimization and LM Backpropagation

The FA-LM optimization algorithm is employed in this study as the training algorithm. FA-LM is a combination of FA and LM backpropagation algorithms. The FA metaheuristic algorithm plays the role as the main optimization method. Based on the initially created population, this algorithm guides the population of ANN model structures to better solutions. Since the problem of constructing an ANN model from a data set is highly complex and features many local minima [53], the application of FA as metaheuristic approach can help the training process to avoid local convergence and reduce the possibility of local traps. It is noted that the LM algorithm has been implemented via the help of the MATLAB’s Statistics and Machine Learning Toolbox [77]. In addition, the FA and the hybrid FA-LM algorithms have been programmed in MATLAB by the authors.

**Figure 9:**
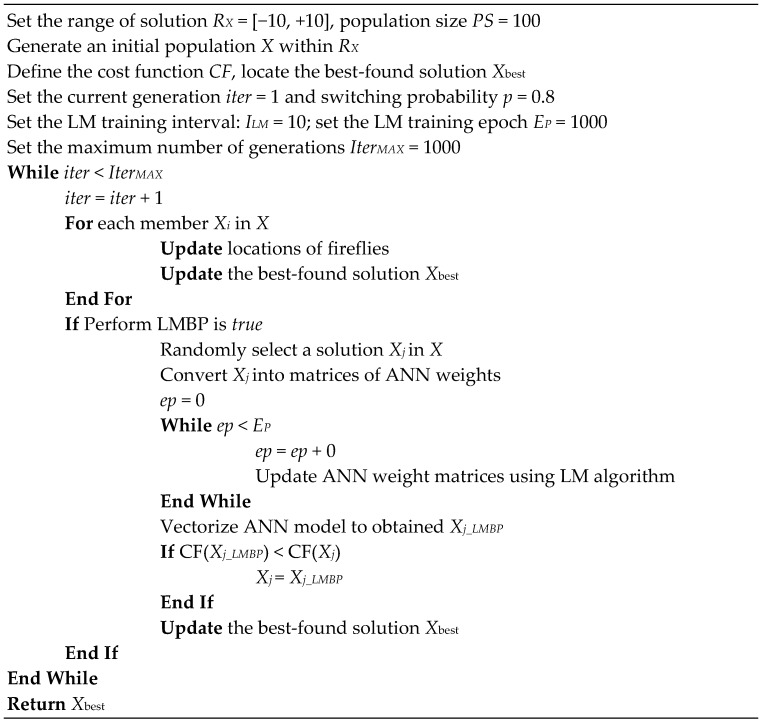
The prosed hybrid FA-LM algorithm for training the ANN model.

In addition, the LM backpropagation is used as a local search method at certain generations during the FA optimization process. Aiming at accelerating the optimization process as well as preventing the stagnation of the FA’s population, the backpropagation with LM algorithm is performed with a randomly selected solution once in 10 generations. This integrated algorithm of FA-LM is illustrated via the pseudo code given in Figure 9. It is noted that the population size of the FA is 100 and the search domain of [−10, 10]. The population is then optimized by the FA-LM algorithm with the maximum number of generation (*G_MAX_*) = 1000. The LM backpropagation is performed with a randomly selected member of the current population. For reducing the computational expense, the LM backpropagation is activated one times in 10 generations. The number of backpropagation training epochs is 1000 and the learning rate used is 0.01, respectively. After being the FA-LM optimization process is accomplished, the trained ANN model is ready for the task of spatial prediction of flash flood occurrences.

## 5. Results and Discussion

### 5.1. Training Results and Performance Assessment

As mentioned earlier, the dataset consisting of 1308 samples is used to construct and verify the ANN based flash flood susceptibility prediction model. This data set is randomly divided into two separated groups: data for model construction (70%) and data for testing (30%) [16,20,31,78,79]. The first group is further partition into two subsets of the training set (80% of the model construction samples) and the validating set (20% of the model construction samples), respectively. Moreover, it is noted that the 12 flood influencing factors have been converted from categorical classes (shown in Figure 7) into continuous values within the range of 0.01 and 0.99 using the approaches described in Tien Bui, et al. [80]. The process of this data conversion process is to fend off the situation where large values of flash-flood conditioning factors dominate other with small values. Accordingly, the statistical description of the flash flood influencing factors is provided in Table 2.

It is also worth noticing that to further facilitate the training phase of ANN, the data set is then normalized by the *Z*-score transformation [81]. The formula of the *Z*-score transformation is described in the following equation:

(10) $$IF_{N}=\frac{IF_{O}-m_{IF}}{s_{IF}},$$

where *IF_N_* and *IF_O_* denotes the normalized and the original influencing factor (IF), respectively. *m_IF_* and *s_IF_* are the mean value and the standard deviation of the IF, respectively.

Additionally, to compute the predictive performance of the flash-flood model, the classification accuracy rate (CAR) for class *i* is calculated using Equation (11):

(11) $$CAR_{i}=\frac{R_{C}^{i}}{R_{A}^{i}}\times 100(\%)\;$$

where $R_{C}^{i}$ and $R_{A}^{i}$ are the number of samples in class *i*-th being categorized correctly and the total number of samples in class *i*-th, respectively. It is worth reminding that there are two class labels, flash flood and non-flash flood.

Performance of the flash-flood models, beside CAR, other statistical metrics can be used i.e., true positive rate (TPR), false positive rate (FPR), false negative rate (FNR), and true negative rate (TNR) [82,83]:

(12) $$TPR=\frac{TP}{TP+FN};\;FPR=\frac{FP}{FP+TN};\;FNR=\frac{FN}{TP+FN};\;TNR=\frac{TN}{TN+FP},$$

where *TP* is true positive; *TN* is true negative; *FP* is false positive, and *FN* is false negative.

In addition, the precision and recall, which are computed using Equations (13) and (14) below, can be used:

(13) $$\mathrm{Precision}=\;\frac{TP}{TP+FP},$$

(14) $$\mathrm{Recall}\;=\;\frac{TP}{TP+FN},$$

In addition to the above performance measurement indices, the Receiver Operating Characteristic (ROC) curve [84] is also used to summary the overall performance of the flash-flood model and a better model is characterized by a high value of AUC.

**Figure 10:**
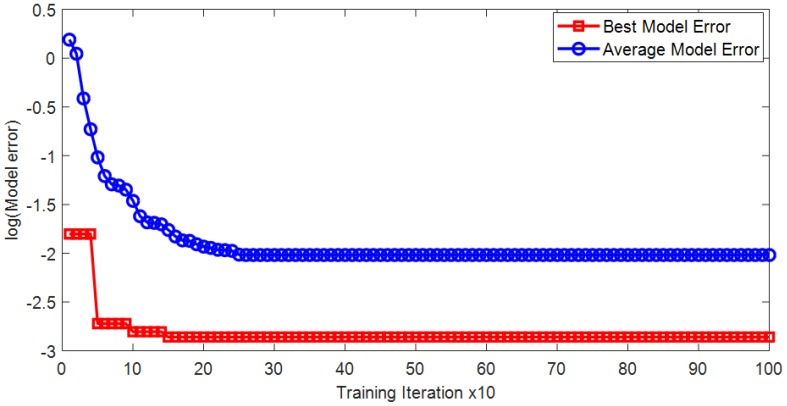
Optimization process of the proposed hybridization of FA and LM.

**Figure 11:**
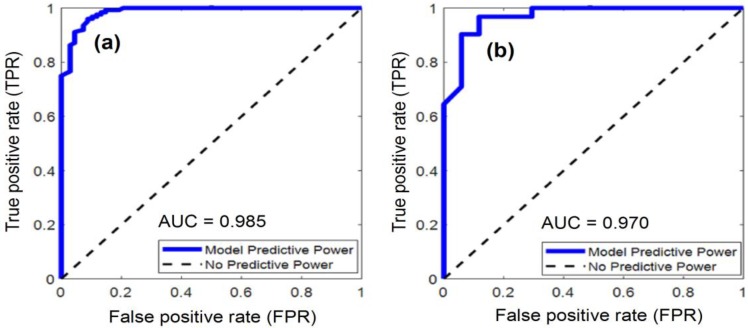
ROCs of the proposed FA-LM-ANN model: (**a**) training phase; (**b**) testing phase.

The optimization process of the hybrid algorithm of FA and LM is illustrated in Figure 10. It can be seen from the figure that the proposed training algorithm can help the ANN model to converge quickly within the allowable number of optimization iteration. The predictive performance of the proposed FA-LM-ANN model is reported in Table 3. It can be seen that the FA-LM-ANN model has obtained good performances in both training (CAR = 92.188% and AUC = 0.985) and testing phase (CAR = 93.750% and AUC = 0.970). The model also achieves desiring values of Precision (0.938) and Recall (0.968) in the testing phase. The ROCs of the FA-LM-ANN are illustrated in Figure 11.

**Figure 12:**
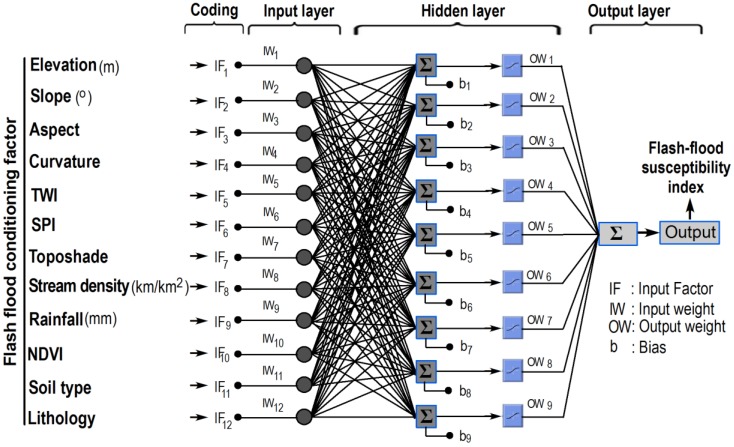
The final trained FA-LM-ANN model for flash-flood susceptibility mapping in this study.

**Figure 13:**
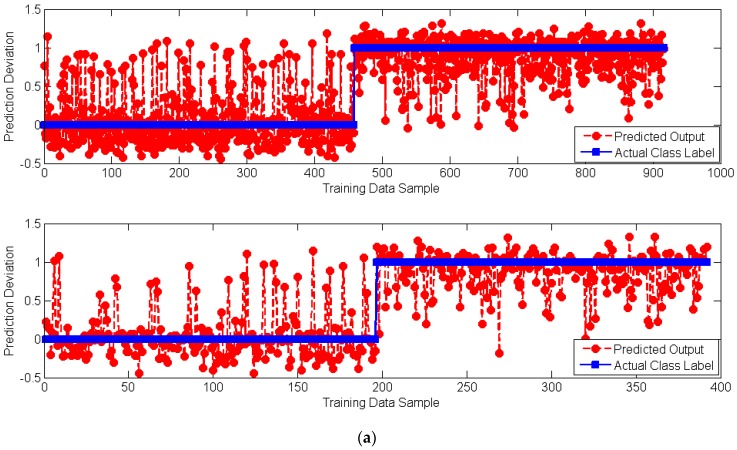

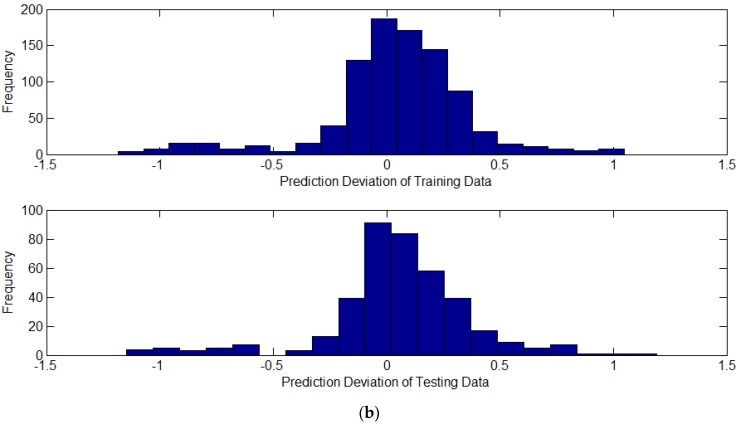
Details of the predicted and actual output data: (**a**) Prediction deviation and (**b**) Prediction Error Distribution.

The final trained FA-LM-ANN model in this research is shown in Figure 12, where the total of 137 weight parameters have been searched and optimized using the proposed FA-LM algorithm. In addition, details of the predicted and actual output data in both the training and testing sets are illustrated in Figure 13. To simplify the presentation of the figure, the class labels of non-flood and flood have been encoded as 0 and 1, respectively. The mean and the standard deviation of the prediction deviation of the data in the training set are 0.039 and 0.320, respectively. For the data in the testing set, the mean and the standard deviation of the prediction deviation are 0.050 and 0.324, respectively.

### 5.2. Model Comparison

For the purpose of result comparison, the performance of the proposed FA-LM ANN is benchmarked against those of the LM-ANN, FA-ANN, support vector machine (SVM) and classification tree (CT). The reason for selecting these models is that both SVM and CT have been successfully employed in flood susceptibility assessment [16,20,38,39,84] and other natural hazards such as landslides [36,38,85,86,87]. These benchmark models are implemented in MATLAB environment via the Statistics and Machine Learning Toolbox [77]. The methods of ANN trained with the conventional backpropagation algorithm are employed in spatial prediction of natural hazards [37,39,88]. In addition, by comparing the performances of the ANN trained with the metaheuristic approach of FA and the proposed FA-LM ANN can help to point out the advantage of the new hybrid ANN’s training algorithm.

To employ the LM-ANN, FA-ANN, SVM, and CT models, it is necessary to select their tuning parameters. In this section, the tuning parameters that lead to the best testing performance of models are selected. For the DT model, the minimal number of observations per tree leaf is selected to 1 as per default settings in MATLAB toolbox [77]. The crucial parameter of LM-ANN and FA-ANN is *Nr* (the number of hidden neurons). In the experiment, as suggested by Heaton [76], this parameter of these two ANN models is set to be 9 which is equal to *Nr* of the proposed FA-LM-ANN. In addition, the maximum number of training epochs = 5000 is used to train the LM-ANN model and the FA-ANN is optimized with a maximum number of iteration = 1000. For the SVM model, the regularization parameter and the RBF kernel parameter are selected based on the grid search as explained in Hoang and Bui [89].

**Figure 14:**
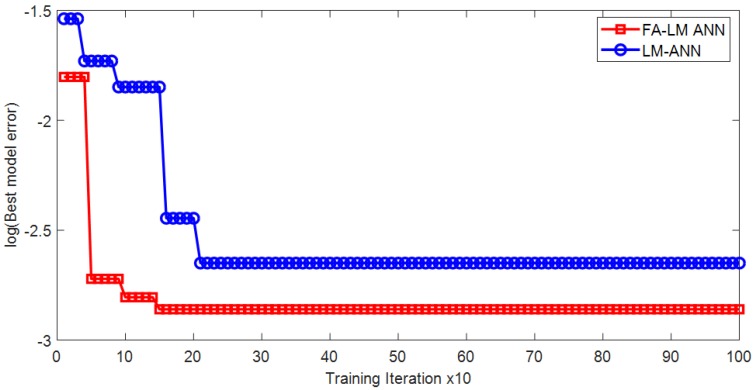
Comparison of convergence rates between FA-LM ANN and LM-ANN.

The prediction results of the prediction models are summarized in Table 4. Considering the model performances in the testing phase, the proposed FA-LM-ANN model has achieved the highest values of CAR (93.750%), AUC (0.970), Precision (0.938), and Recall (0.968). The second-best model is SVM with CAR = 91.667%, AUC = 0.960, Precision = 0.909, and Recall = 0.968, followed by FA-ANN, CT, and LM-ANN. It can be noticed that there is an improvement in CAR when the ANN model is trained by the FA algorithm (91.667%) instead of the LM backpropagation (88.931%); however, the AUC value of the first approach (0.917) is worse than that of the second approach (0.937). In addition, Figure 14 provides the comparison of the convergence rates between the two ANN training approaches of FA-LM and LM. It can be observed from this figure that the convergence of the model training phase performed by FA-LM is faster than that performed by LM.

To further confirm the predictive capability of the proposed model, a ten-fold cross validation process is also performed in this section. Using the cross validation process, the training and testing phase of the prediction models are carried out 10 times. In each time, 90% of the data set is employed for model construction; 10% of the data set is reserved for model testing. The experimental outcomes are reported in Table 5 which shows the mean and the standard deviation (Std.) of the flash flood susceptibility classification results. It can be observed that the proposed FA-LM ANN has achieved the highest average predictive performance in terms of CAR = 90.137% and AUC = 0.970. This outcome is clearly better than those of LM-ANN (CAR = 88.154% and AUC = 0.926), FA-ANN (CAR = 89.308% and AUC = 0.919), SVM (CAR = 87.923% and AUC = 0.929), and CT (CAR = 87.077% and AUC = 0.908). Overall, comparing with FA-ANN and LM-ANN, there are significant enhancements in terms of both CAR and AUC when the ANN is constructed by means of the hybrid FA-LM approach.

### 5.3. Establishment of the Flash Flood Susceptibility Map

Because both the training and testing results have pointed out that FA-LM-ANN is the best model for the dataset collected in the BHBY area, the model is then employed to compute the flash-flood susceptibility for each of all the pixels in the study area.

**Figure 15:**
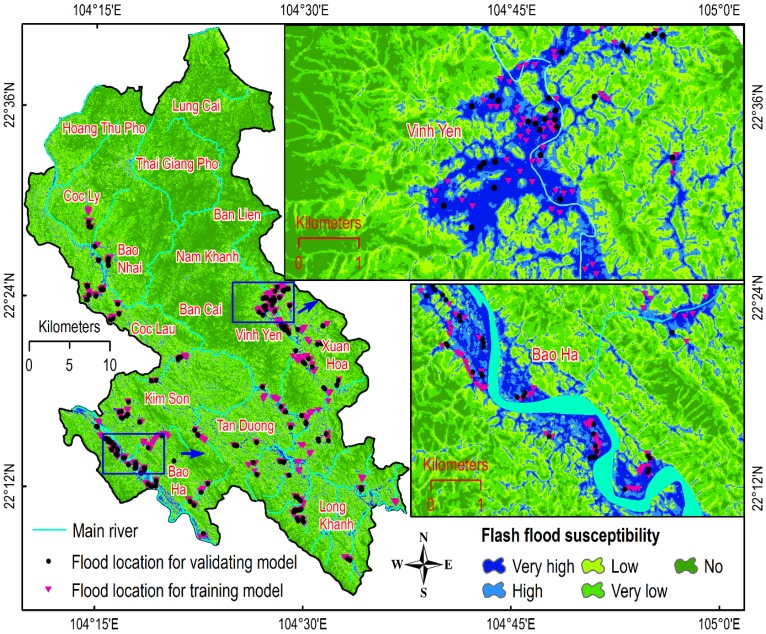
Flash flood susceptibility map for the study area.

The predictive results of flash flood susceptibility are transformed to a grid format using the python tool (mentioned in Section 3.2) and open in ArcGIS 10.4 software (ESRI Inc., Redlands, CA, USA). Based on these computed indices, the flash-flood susceptibility map (see Figure 15) was obtained and visualized by mean of five classes: very high, high, low, very low, and no. The thresholds for dividing these computed indices into the five classes were determined by using the natural break classification method [90].

Interpretation of the flash-flood susceptibility map shows that all flash flood locations are located in the two classes, very high and high, indicating that that the proposed FA-LM-ANN model has successfully determined flash flood prone areas.

## 6. Conclusions

This research proposes a new methodology using Sentinel-1 SAR imagery and machine learning techniques for spatial prediction of flash flood hazards. The SAR imagery was used to detect flash flood locations, whereas the proposed FA-LM-ANN was used to establish the flash flood prediction model. The methodology was applied for the Bac Ha Bao Yen (BHBY) area, a most flood-prone area in Vietnam. Accordingly, the GIS database was established containing the information regarding historical cases of flash flood events and 12 flood-conditioning factors.

The advantage of the Sentinel-1 SAR imagery with the change detection method is the ability to capture and detect flash flood areas with high accuracy. However, flash floods often occur in a short time; therefore, this method is feasible for flash flood mapping if the Sentinel sensor captures the images at the time of flash flood occurrence. Regarding the proposed FA-LM-ANN, this artificial intelligence model is capable to meliorate the model performance. This is because FA is employed as a swarm intelligence method to optimize the parameter of ANN so that a decision boundary for classification of non-flood and flood locations can be identified accurately, whereas LM backpropagation serves as a local search method to increase the convergence of the swarm intelligence-based training algorithm.

Because the proposed FA-LM-ANN is constructed with 12 input neurons, nine hidden neurons, and one output neuron, which results in 119 weights, therefore, the search space of the FA has 119 dimensions. In other words, the coordination of each firefly consists of 119 parameters. The swarm of 100 fireflies was used with 1000 running iterations have resulted in 100,000 searches for possible combinations the weighs of the FA-LM-ANN model. Consequently, the high prediction capability of the proposed flash-flood model indicates that the hybridization of FA—a metaheuristic algorithm and the LM backpropagation has trained the model successfully.

Compared to benchmarks like LM-ANN, FA-ANN, SVM, and DT, the prediction result of the proposed model is better; therefore, it can be concluded that the proposed FA-LM ANN is a very promising tool to assist decision makers, especially local authorities, in developing effective flash flood countermeasures and land-use planning. Future extensions of the current study may include applying the newly constructed model for predicting flood risks in other study areas and enhancing the learning capability of the proposed model with other metaheuristic optimization algorithms.

## Figures and Tables

**Table 1:** Sentinel-1A SAR images used for flash flood detection.

Date of Acquisition	Mode	Polarization Used	Relative Orbit	Pass Direction	Note
23 July 2017	IW	VV	26	Ascending	Pre-event
04 August 2017	IW	VV	26	Ascending	Post-event
30 July 2017	IW	VV	128	Ascending	Pre-event
10 October 2017	IW	VV	128	Ascending	Post-event

**Table 2:** Statistical description of the collected data.

Influencing Factor	Min	Mean	Median	Standard Deviation	Skewness	Max
IF1	0.010	0.165	0.010	0.257	1.747	0.990
IF2	0.010	0.248	0.120	0.286	0.806	0.990
IF3	0.100	0.594	0.620	0.262	0.118	0.990
IF4	0.010	0.479	0.500	0.180	0.606	0.990
IF5	0.010	0.601	0.660	0.308	0.329	0.990
IF6	0.010	0.200	0.170	0.228	1.074	0.990
IF7	0.010	0.213	0.010	0.256	0.842	0.990
IF8	0.010	0.416	0.340	0.282	0.240	0.990
IF9	0.010	0.428	0.400	0.301	0.063	0.990
IF10	0.010	0.553	0.570	0.264	0.491	0.990
IF11	0.010	0.273	0.170	0.208	1.660	0.990
IF12	0.010	0.294	0.160	0.285	0.847	0.990

**Table 3:** Prediction performance of the FA-LM ANN model.

Phases	Performance Measurement Indices							
	CAR (%)	AUC	TPR	FPR	FNR	TNR	Precision	Recall
Training phase	92.188	0.985	0.976	0.177	0.024	0.824	0.910	0.976
Testing phase	93.750	0.970	0.968	0.118	0.032	0.882	0.938	0.968

**Table 4:** Result comparison.

Performances	Prediction Models				
	FA-LM ANN	LM-ANN	FA-ANN	SVM	CT
*Training Phase*					
CAR (%)	93.750	92.639	94.792	92.708	98.958
AUC	0.986	0.957	0.972	0.984	0.999
TPR	0.984	0.973	0.960	0.992	1.000
FPR	0.147	0.121	0.074	0.191	0.029
FNR	0.016	0.027	0.040	0.008	0.000
TNR	0.853	0.880	0.927	0.809	0.971
Precision	0.924	0.890	0.960	0.904	0.984
Recall	0.984	0.973	0.960	0.992	1.000
*Testing Phase*					
CAR (%)	93.750	88.931	91.667	91.667	89.583
AUC	0.970	0.937	0.917	0.960	0.904
TPR	0.968	0.924	0.936	0.968	0.936
FPR	0.118	0.145	0.118	0.177	0.177
FNR	0.032	0.076	0.065	0.032	0.065
TNR	0.882	0.855	0.882	0.824	0.824
Precision	0.938	0.864	0.936	0.909	0.906
Recall	0.968	0.924	0.936	0.968	0.936

**Table 5:** Result of the 10-fold cross validation process.

Performance	Prediction Models									
	FA-LM ANN		LM-ANN		FA-ANN		SVM		CT	
	*Mean*	*Std.*	*Mean*	*Std.*	*Mean*	*Std.*	*Mean*	*Std.*	*Mean*	*Std.*
CAR (%)	90.137	2.614	88.154	2.383	89.308	2.034	87.923	1.851	87.077	2.372
AUC	0.970	0.016	0.926	0.022	0.919	0.029	0.929	0.016	0.908	0.032
TPR	0.945	0.033	0.962	0.032	0.959	0.018	0.926	0.028	0.902	0.023
FPR	0.165	0.065	0.199	0.052	0.172	0.050	0.168	0.037	0.160	0.048
FNR	0.056	0.015	0.039	0.011	0.042	0.009	0.074	0.001	0.099	0.006
TNR	0.835	0.065	0.802	0.052	0.828	0.050	0.832	0.037	0.840	0.048
Precision	0.914	0.030	0.831	0.035	0.849	0.036	0.848	0.027	0.851	0.036
Recall	0.945	0.033	0.962	0.032	0.959	0.018	0.926	0.028	0.902	0.023

## References

[1] Siahkamari S., Haghizadeh A., Zeinivand H., Tahmasebipour N., Rahmati O. (2018). Spatial prediction of flood-susceptible areas using frequency ratio and maximum entropy models. Geocarto Int..

[2] Woodruff S.C., Regan P. (2018). Quality of national adaptation plans and opportunities for improvement. Mitig. Adapt. Strat. Glob. Chang..

[3] National Weather Service (NWS) (2018). What Is Flash Flooding. https://www.weather.gov/phi/FlashFloodingDefinition.

[4] Archer D.R., Fowler H.J. (2018). Characterising flash flood response to intense rainfall and impacts using historical information and gauged data in Britain. J. Flood Risk Manag..

[5] Gourley J.J., Flamig Z.L., Vergara H., Kirstetter P.E., Clark R.A., Argyle E., Arthur A., Martinaitis S., Terti G., Erlingis J.M. (2017). The FLASH Project: Improving the Tools for Flash Flood Monitoring and Prediction across the United States. Bull. Am. Meteorol. Soc..

[6] Papagiannaki K., Kotroni V., Lagouvardos K., Bezes A. (2017). Flash Flood Risk and Vulnerability Analysis in Urban Areas: The Case of October 22, 2015, in Attica, Greece. Perspectives on Atmospheric Sciences.

[7] Lucía A., Schwientek M., Eberle J., Zarfl C. (2018). Planform changes and large wood dynamics in two torrents during a severe flash flood in Braunsbach, Germany 2016. Sci. Total Environ..

[8] He B., Huang X., Ma M., Chang Q., Tu Y., Li Q., Zhang K., Hong Y. (2018). Analysis of flash flood disaster characteristics in china from 2011 to 2015. Nat. Hazards.

[9] Faccini F., Luino F., Sacchini A., Turconi L. (2015). Flash flood events and urban development in Genoa (Italy): Lost in translation. Engineering Geology for Society and Territory.

[10] Nguyen H., Degener J., Kappas M. (2015). Flash Flood Prediction by Coupling KINEROS2 and HEC-RAS Models for Tropical Regions of Northern Vietnam. Hydrology.

[11] Yates D.N., Warner T.T., Leavesley G.H. (2000). Prediction of a Flash Flood in Complex Terrain. Part II: A Comparison of Flood Discharge Simulations Using Rainfall Input from Radar, a Dynamic Model, and an Automated Algorithmic System. J. Appl. Meteorol..

[12] Volkmann T.H.M., Lyon S.W., Gupta H.V., Troch P.A. (2010). Multicriteria design of rain gauge networks for flash flood prediction in semiarid catchments with complex terrain. Water Resour. Res..

[13] El Kadi Abderrezzak K., Paquier A., Mignot E. (2009). Modelling flash flood propagation in urban areas using a two-dimensional numerical model. Nat. Hazards.

[14] Liu W.C., Wu C.Y. (2011). Flash flood routing modeling for levee-breaks and overbank flows due to typhoon events in a complicated river system. Nat. Hazards.

[15] Tien Bui D., Pradhan B., Nampak H., Bui Q.-T., Tran Q.-A., Nguyen Q.-P. (2016). Hybrid artificial intelligence approach based on neural fuzzy inference model and metaheuristic optimization for flood susceptibilitgy modeling in a high-frequency tropical cyclone area using GIS. J. Hydrol..

[16] Khosravi K., Pham B.T., Chapi K., Shirzadi A., Shahabi H., Revhaug I., Prakash I., Tien Bui D. (2018). A comparative assessment of decision trees algorithms for flash flood susceptibility modeling at Haraz watershed, northern Iran. Sci. Total. Environ..

[17] Ahmadlou M., Karimi M., Alizadeh S., Shirzadi A., Parvinnejhad D., Shahabi H., Panahi M. (2018). Flood susceptibility assessment using integration of adaptive network-based fuzzy inference system (ANFIS) and biogeography-based optimization (BBO) and BAT algorithms (BA). Geocarto Int..

[18] Tzavella K., Fekete A., Fiedrich F. (2018). Opportunities provided by geographic information systems and volunteered geographic information for a timely emergency response during flood events in Cologne, Germany. Nat. Hazards.

[19] Ahmed N., Atta-ur-Rahman Dash S., Mahmud M. (2018). Flood-Prediction Techniques Based on Geographical Information System Using Wireless Sensor Networks. Advances in Data and Information Sciences.

[20] Tehrany M.S., Pradhan B., Mansor S., Ahmad N. (2015). Flood susceptibility assessment using GIS-based support vector machine model with different kernel types. CATENA.

[21] Li Y., Martinis S., Plank S., Ludwig R. (2018). An automatic change detection approach for rapid flood mapping in Sentinel-1 SAR data. Int. J. Appl. Earth Obs. Geoinf..

[22] Amitrano D., Di Martino G., Iodice A., Riccio D., Ruello G. (2018). Unsupervised Rapid Flood Mapping Using Sentinel-1 GRD SAR Images. IEEE Trans. Geosci. Remote Sens..

[23] Al-Abadi A.M. (2018). Mapping flood susceptibility in an arid region of southern Iraq using ensemble machine learning classifiers: A comparative study. Arab. J. Geosci..

[24] Li H., Zhang Z., Liu Z. (2017). Application of artificial neural networks for catalysis: A review. Catalysts.

[25] Dudley J.J., Kristensson P.O. (2018). A Review of User Interface Design for Interactive Machine Learning. ACM Trans. Interact. Intell. Syst. (TiiS).

[26] Nandi A., Mandal A., Wilson M., Smith D. (2016). Flood hazard mapping in Jamaica using principal component analysis and logistic regression. Environ. Earth Sci..

[27] Khosravi K., Nohani E., Maroufinia E., Pourghasemi H.R. (2016). A GIS-based flood susceptibility assessment and its mapping in Iran: A comparison between frequency ratio and weights-of-evidence bivariate statistical models with multi-criteria decision-making technique. Nat. Hazards.

[28] Razavi Termeh S.V., Kornejady A., Pourghasemi H.R., Keesstra S. (2018). Flood susceptibility mapping using novel ensembles of adaptive neuro fuzzy inference system and metaheuristic algorithms. Sci. Total Environ..

[29] Lee S., Kim J.C., Jung H.S., Lee M.J., Lee S. (2017). Spatial prediction of flood susceptibility using random-forest and boosted-tree models in Seoul metropolitan city, Korea. Geomat. Nat. Hazards Risk.

[30] Tien Bui D., Hoang N.D. (2017). A Bayesian framework based on a Gaussian mixture model and radial-basis-function Fisher discriminant analysis (BayGmmKda V1.1) for spatial prediction of floods. Geosci. Model. Dev..

[31] Chapi K., Singh V.P., Shirzadi A., Shahabi H., Bui D.T., Pham B.T., Khosravi K. (2017). A novel hybrid artificial intelligence approach for flood susceptibility assessment. Environ. Model. Softw..

[32] Sachdeva S., Bhatia T., Verma A.K. Flood susceptibility mapping using GIS-based support vector machine and particle swarm optimization: A case study in Uttarakhand (India). Proceedings of the 2017 8th International Conference on Computing, Communication and Networking Technologies (ICCCNT).

[33] Rahmati O., Pourghasemi H.R. (2017). Identification of Critical Flood Prone Areas in Data-Scarce and Ungauged Regions: A Comparison of Three Data Mining Models. Water Resour. Manag..

[34] Youssef A.M., Pradhan B., Hassan A.M. (2011). Flash flood risk estimation along the St. Katherine road, southern Sinai, Egypt using GIS based morphometry and satellite imagery. Environ. Earth Sci..

[35] Sahoo G.B., Ray C., De Carlo E.H. (2006). Use of neural network to predict flash flood and attendant water qualities of a mountainous stream on Oahu, Hawaii. J. Hydrol..

[36] Pham B.T., Tien Bui D., Pourghasemi H.R., Indra P., Dholakia M.B. (2017). Landslide susceptibility assesssment in the Uttarakhand area (India) using GIS: A comparison study of prediction capability of naïve bayes, multilayer perceptron neural networks, and functional trees methods. Theor. Appl. Clim..

[37] Hoang N.D., Tien Bui D. (2017). GIS-Based Landslide Spatial Modeling Using Batch-Training Back-propagation Artificial Neural Network: A Study of Model Parameters. Advances and Applications in Geospatial Technology and Earth Resources.

[38] Kalantar B., Pradhan B., Naghibi S.A., Motevalli A., Mansor S. (2018). Assessment of the effects of training data selection on the landslide susceptibility mapping: A comparison between support vector machine (SVM), logistic regression (LR) and artificial neural networks (ANN). Geomat. Nat. Hazards Risk.

[39] Aditian A., Kubota T., Shinohara Y. (2018). Comparison of GIS-based landslide susceptibility models using frequency ratio, logistic regression, and artificial neural network in a tertiary region of Ambon, Indonesia. Geomorphology.

[40] Yaghini M., Khoshraftar M.M., Fallahi M. (2013). A hybrid algorithm for artificial neural network training. Eng. Appl. Artif. Intell..

[41] Ghasemiyeh R., Moghdani R., Sana S.S. (2017). A Hybrid Artificial Neural Network with Metaheuristic Algorithms for Predicting Stock Price. Cybern. Syst..

[42] Kuok K.K., Kueh S.M., Chiu P.C. (2018). Bat optimisation neural networks for rainfall forecasting: Case study for Kuching city. J. Water Clim. Chang..

[43] Faris H., Aljarah I., Mirjalili S. (2018). Improved monarch butterfly optimization for unconstrained global search and neural network training. Appl. Intell..

[44] Soodi H.A., Vural A.M. (2018). STATCOM Estimation Using Back-Propagation, PSO, Shuffled Frog Leap Algorithm, and Genetic Algorithm Based Neural Networks. Comput. Intell. Neurosci..

[45] Jaddi N.S., Abdullah S. (2018). Optimization of neural network using kidney-inspired algorithm with control of filtration rate and chaotic map for real-world rainfall forecasting. Eng. Appl. Artif. Intell..

[46] Hacibeyoglu M., Ibrahim M.H. (2018). A Novel Multimean Particle Swarm Optimization Algorithm for Nonlinear Continuous Optimization: Application to Feed-Forward Neural Network Training. Sci. Program..

[47] Ojha V.K., Abraham A., Snášel V. (2017). Metaheuristic design of feedforward neural networks: A review of two decades of research. Eng. Appl. Artif. Intell..

[48] Vnexpress (2017). Flash Floods Kill 18, Isolate Towns in Northern Vietnam. VnExpress.net.

[49] Borga M., Anagnostou E.N., Blöschl G., Creutin J.D. (2011). Flash flood forecasting, warning and risk management: The HYDRATE project. Environ. Sci. Policy.

[50] Dai K., Li Z., Tomás R., Liu G., Yu B., Wang X., Cheng H., Chen J., Stockamp J. (2016). Monitoring activity at the Daguangbao mega-landslide (China) using Sentinel-1 TOPS time series interferometry. Remote Sens. Environ..

[51] Clement M., Kilsby C., Moore P. (2018). Multi-temporal synthetic aperture radar flood mapping using change detection. J. Flood Risk Manag..

[52] Twele A., Cao W., Plank S., Martinis S. (2016). Sentinel-1-based flood mapping: A fully automated processing chain. Int. J. Remote Sens..

[53] Lee J.-S. (1980). Digital image enhancement and noise filtering by use of local statistics. IEEE Trans. Pattern Anal. Mach. Intell..

[54] Basheer I.A., Hajmeer M. (2000). Artificial neural networks: Fundamentals, computing, design, and application. J. Microbiol. Methods.

[55] Tran T.H., Hoang N.D. (2016). Predicting Colonization Growth of Algae on Mortar Surface with Artificial Neural Network. J. Comput. Civ. Eng..

[56] Rumelhart D.E., Hinton G.E., Williams R.J. (1986). Learning representations by back-propagating errors. Nature.

[57] Hagan M.T., Menhaj M.B. (1994). Training feedforward networks with the Marquardt algorithm. IEEE Trans. Neural Netw..

[58] Reyes J., Morales-Esteban A., Martínez-Álvarez F. (2013). Neural networks to predict earthquakes in Chile. Appl. Soft Comput..

[59] Beale M.H., Hagan M.T., Demuth H.B. (2018). Neural Network Toolbox User’s Guide.

[60] Yang X.S. (2010). Firefly algorithm, stochastic test functions and design optimisation. Int. J. Bio-Inspired Comput..

[61] Fister I., Fister I., Yang X.S., Brest J. (2013). A comprehensive review of firefly algorithms. Swarm Evol. Comput..

[62] Bui D.K., Nguyen T., Chou J.S., Nguyen-Xuan H., Ngo T.D. (2018). A modified firefly algorithm-artificial neural network expert system for predicting compressive and tensile strength of high-performance concrete. Constr. Build. Mater..

[63] Wang D., Luo H., Grunder O., Lin Y., Guo H. (2017). Multi-step ahead electricity price forecasting using a hybrid model based on two-layer decomposition technique and BP neural network optimized by firefly algorithm. Appl. Energy.

[64] Cheng M.Y., Hoang N.D. (2017). Estimating construction duration of diaphragm wall using firefly-tuned least squares support vector machine. Neural Comput. Appl..

[65] Tilahun S.L., Ngnotchouye J.M.T., Hamadneh N.N. (2017). Continuous versions of firefly algorithm: A review. Artif. Intell. Rev..

[66] Qi C., Fourie A., Zhao X. (2018). Back-Analysis Method for Stope Displacements Using Gradient-Boosted Regression Tree and Firefly Algorithm. J. Comput. Civ. Eng..

[67] Hou L., Zhao C., Wu C., Moon S., Wang X. (2017). Discrete Firefly Algorithm for Scaffolding Construction Scheduling. J. Comput. Civ. Eng..

[68] Yang X.S. (2009). Firefly algorithms for multimodal optimization. Stochastic Algorithms: Foundations and Applications, Proceedings of the International Symposium on Stochastic Algorithms, Sapporo, Japan, 26–28 October 2009.

[69] GSO (2017). Lao Cai Statistical Year Book 2016.

[70] Le T.P.Q., Garnier J., Gilles B., Sylvain T., Van Minh C. (2007). The changing flow regime and sediment load of the Red River, Viet Nam. J. Hydrol..

[71] Shafizadeh-Moghadam H., Valavi R., Shahabi H., Chapi K., Shirzadi A. (2018). Novel forecasting approaches using combination of machine learning and statistical models for flood susceptibility mapping. J. Environ. Manag..

[72] USGS (2017). The United States Geological Survey Earth Resources Observation and Science Cente. http://earthexplorer.usgs.gov.

[73] Martinović K., Gavin K., Reale C. (2016). Development of a landslide susceptibility assessment for a rail network. Eng. Geol..

[74] Bai S., Wang J., Lu G., Zhou P., Hou S., Xu S. (2010). GIS-based logistic regression for landslide susceptibility mapping of the Zhongxian segment in the Three Gorges area, China. Geomorphology.

[75] Ayalew L., Yamagishi H. (2005). The application of GIS-based logistic regression for landslide susceptibility mapping in the Kakuda-Yahiko Mountains, Central Japan. Geomorphology.

[76] Heaton J. (2008). Introduction to Neural Networks for C#.

[77] Matwork (2017). Statistics and Machine Learning Toolbox User’s Guide.

[78] Tehrany M.S., Pradhan B., Jebur M.N. (2014). Flood susceptibility mapping using a novel ensemble weights-of-evidence and support vector machine models in GIS. J. Hydrol..

[79] Hong H., Panahi M., Shirzadi A., Ma T., Liu J., Zhu A.X., Chen W., Kougias I., Kazakis N. (2018). Flood susceptibility assessment in Hengfeng area coupling adaptive neuro-fuzzy inference system with genetic algorithm and differential evolution. Sci. Total. Environ..

[80] Tien Bui D., Tran A.T., Klempe H., Pradhan B., Revhaug I. (2016). Spatial prediction models for shallow landslide hazards: A comparative assessment of the efficacy of support vector machines, artificial neural networks, kernel logistic regression, and logistic model tree. Landslides.

[81] Montavon G., Orr G., Müller K.R. (2012). Neural Networks: Tricks of the Trade.

[82] Asencio-Cortés G., Martínez-Álvarez F., Troncoso A., Morales-Esteban A. (2017). Medium–large earthquake magnitude prediction in Tokyo with artificial neural networks. Neural Comput. Appl..

[83] Martínez-Álvarez F., Reyes J., Morales-Esteban A., Rubio-Escudero C. (2013). Determining the best set of seismicity indicators to predict earthquakes. Two case studies: Chile and the Iberian Peninsula. Knowl.-Based Syst..

[84] Van Erkel A.R., Pattynama P.M.T. (1998). Receiver operating characteristic (ROC) analysis: Basic principles and applications in radiology. Eur. J. Radiol..

[85] Pham B.T., Jaafari A., Prakash I., Bui D.T. (2018). A novel hybrid intelligent model of support vector machines and the MultiBoost ensemble for landslide susceptibility modeling. Bull. Eng. Geol. Environ..

[86] Hong H., Liu J., Bui D.T., Pradhan B., Acharya T.D., Pham B.T., Zhu A.X., Chen W., Ahmad B.B. (2018). Landslide susceptibility mapping using J48 Decision Tree with AdaBoost, Bagging and Rotation Forest ensembles in the Guangchang area (China). CATENA.

[87] Chen W., Xie X., Wang J., Pradhan B., Hong H., Bui D.T., Duan Z., Ma J. (2017). A comparative study of logistic model tree, random forest, and classification and regression tree models for spatial prediction of landslide susceptibility. CATENA.

[88] Satir O., Berberoglu S., Donmez C. (2016). Mapping regional forest fire probability using artificial neural network model in a Mediterranean forest ecosystem. Geomat. Nat. Hazards Risk.

[89] Hoang N.D., Bui D.T. (2018). Predicting earthquake-induced soil liquefaction based on a hybridization of kernel Fisher discriminant analysis and a least squares support vector machine: A multi-dataset study. Bull. Eng. Geol. Environ..

[90] Bednarik M., Magulová B., Matys M., Marschalko M. (2010). Landslide susceptibility assessment of the Kraľovany–Liptovský Mikuláš railway case study. Phys. Chem. Earth Parts A B C.

